# Identification of MTMR2 as an AML-associated candidate biomarker derived from lipid metabolism–related transcriptomic analysis

**DOI:** 10.3389/fonc.2026.1837038

**Published:** 2026-05-29

**Authors:** Chenchen Liu, Yueyuan Pan, Minggui Chen, Songyu Li, Chong Zhang

**Affiliations:** 1 Zhanjiang Institute of Clinical Medicine, Zhanjiang Central Hospital, Guangdong Medical University (Central People’s Hospital of Zhanjiang), Zhanjiang, Guangdong, China; 2 Precision Clinical Laboratory, Zhanjiang Central Hospital, Guangdong Medical University (Central People’s Hospital of Zhanjiang), Zhanjiang, Guangdong, China

**Keywords:** acute myeloid leukemia, immune infiltration, lipid metabolism, machine learning, MTMR2, prognosis, ROC, ssGSEA

## Abstract

**Background:**

Acute myeloid leukemia (AML) is a diverse malignant hematologic disorder with poor clinical outcomes. Increasing evidence suggests that metabolic reprogramming, particularly lipid metabolism, contributes to AML progression and may offer new opportunities for biomarker discovery and therapeutic targeting. However, lipid metabolism–related hub genes with diagnostic and prognostic relevance in AML have not been systematically characterized.

**Methods:**

Expression profiles from GSE114868 and GSE9476 were analyzed to identify lipid metabolism-associated differentially expressed genes. Functional enrichment, single-sample gene set enrichment analysis (ssGSEA), weighted gene co-expression network analysis (WGCNA), machine-learning-based feature selection, diagnostic receiver operating characteristic (ROC) analysis, survival analysis, and immune infiltration analysis were performed. MTMR2 expression was further validated by RT-qPCR in an expanded clinical cohort of AML patients and healthy controls, and selected lipid-related clinical parameters were explored.

**Results:**

Lipid metabolism-related pathways were significantly altered in AML samples. Integration of differential expression analysis, WGCNA, LASSO regression, random forest analysis, and SVM-RFE identified MTMR2 as a candidate lipid metabolism-associated biomarker. MTMR2 was markedly upregulated in AML across independent datasets and showed good diagnostic performance. Kaplan-Meier analysis suggested an association between high MTMR2 expression and poorer overall survival. High MTMR2 expression was also associated with immune- and inflammation-related transcriptional features and with altered inferred immune-cell infiltration patterns. RT-qPCR analysis confirmed higher MTMR2 expression in AML samples, and exploratory clinical analysis showed lower ApoA1 and LDL-C levels and higher TG levels in AML patients than in healthy controls.

**Conclusion:**

This study identifies MTMR2 as a lipid metabolism-associated candidate biomarker in AML and provides preliminary clinical evidence supporting its increased expression and association with altered lipid-related parameters. These findings support further mechanistic and clinical validation of MTMR2 in larger independent AML cohorts.

## 1 Introduction

Acute myeloid leukemia (AML) remains a clinically challenging malignancy because biological heterogeneity translates into substantial differences in treatment response, relapse risk, and survival. Although molecular stratification has improved disease classification, clinically useful biomarkers that capture additional dimensions of AML biology are still needed (1).

Metabolic reprogramming has emerged as a key feature of AML biology (2). In addition to altered glucose and amino acid metabolism, increasing attention has focused on lipid metabolism as a key component of leukemic cell survival and adaptation (3). Accumulating evidence suggests that lipid metabolic reprogramming supports AML progression by reshaping membrane lipid composition, modulating signaling pathways, sustaining energy homeostasis, and promoting treatment resistance, particularly in leukemic stem cells (3–7). These observations support the rationale for identifying lipid metabolism–associated biomarkers in AML.

Lipid metabolism in AML may also be associated with immune-related biological processes (3, 8). AML exhibits marked immune dysregulation, and increasing evidence supports a close link between metabolic states and immune regulation (9, 10). This suggests that lipid metabolism–related genes may reflect not only metabolic alterations, but also immune-related transcriptional characteristics in AML (8, 10).

Recent advances in bioinformatics and machine learning have enabled systematic identification of candidate disease biomarkers from high-dimensional transcriptomic datasets. Weighted gene co-expression network analysis (WGCNA), least absolute shrinkage and selection operator (LASSO), random forest, and support vector machine-recursive feature elimination (SVM-RFE) have been widely used to screen robust diagnostic and prognostic genes. Recent studies in other disease contexts further illustrate that integrated transcriptomic analysis combined with machine-learning-based feature selection can support the discovery of disease-associated molecular signatures (11–14). However, few studies have integrated lipid metabolism-related gene signatures with machine learning and clinical validation to identify AML-associated biomarkers.

To screen for lipid metabolism–associated hub genes in AML, we analyzed the GEO datasets GSE114868 and GSE9476 using an integrated workflow that incorporated comparative transcriptomics, ssGSEA, WGCNA, and three machine-learning algorithms. Among the prioritized candidates, MTMR2, a myotubularin-related phosphoinositide 3-phosphatase involved in endosomal membrane homeostasis and trafficking, was selected for further investigation in AML (15–17). We next assessed its diagnostic and prognostic relevance, examined MTMR2-associated biological pathways and immune infiltration patterns, and validated its expression in clinical samples by RT-qPCR. Collectively, these results identify MTMR2 as a lipid metabolism–associated candidate biomarker in AML and provide a rationale for further mechanistic and translational investigation.

## 2 Methods

### 2.1 Data acquisition and preprocessing

Gene expression profiles of AML and healthy control samples were obtained from the Gene Expression Omnibus (GEO) database. The GSE114868 dataset was used as the primary discovery cohort, and the GSE9476 dataset was used for external validation. The GSE12417 cohort was additionally used for exploratory Cox regression analysis. Standard preprocessing procedures were performed, including probe annotation, gene-symbol harmonization, removal of duplicated gene symbols, and within-dataset normalization. Because these datasets were used separately for discovery, validation, and exploratory survival modeling rather than being merged for cross-cohort testing, cross-platform batch correction was not applied. Unless otherwise specified, all bioinformatic analyses were performed using R software (version 4.3.3). The major R packages used in this study included WGCNA (version 1.73), glmnet (version 4.1.10), randomForest (version 4.7.1.2) and GSVA (version 1.50.5).

### 2.2 Expression profiling comparison and functional enrichment

Global expression patterns were first examined by principal component analysis (PCA) with the FactoMineR and factoextra packages. Expression profiles of AML and control samples were compared using the limma package (18). Differentially expressed genes (DEGs) were identified based on |log2FC| > 1 and adjusted P < 0.05. Functional annotation of DEGs, comprising Gene Ontology (GO) and Kyoto Encyclopedia of Genes and Genomes (KEGG) analyses, was executed with the clusterProfiler package (19, 20). Most bioinformatic figures were generated with ggplot2.

### 2.3 Single-sample gene set enrichment analysis

Enrichment scores were quantified with the GSVA package (21, 22) using the ssGSEA approach. The REACTOME_METABOLISM_OF_LIPIDS gene set from the Molecular Signatures Database (MSigDB) was used to estimate lipid metabolism activity. Predefined signatures for 28 immune cell populations were used to infer immune-cell representation across samples. Associations between MTMR2 expression and immune-related ssGSEA scores were examined using Spearman correlation analysis and displayed graphically.

### 2.4 Weighted gene co-expression network analysis

WGCNA (23) was conducted to identify AML-related gene modules. The top 25% of genes ranked by coefficient of variation were selected for network construction. Scale-free topology was achieved using a soft-threshold power (β) of 14. Hierarchical clustering combined with dynamic tree cutting was applied to define co-expression modules. Module eigengenes were subsequently correlated with disease phenotype to identify AML-associated modules. Genes within the AML-associated modules were retained for subsequent intersection analysis using |gene significance (GS)| > 0.2 and |module membership (MM)| > 0.6 as filtering criteria.

### 2.5 Candidate gene selection and machine learning algorithms

Lipid metabolism–related candidate genes were identified by intersecting DEGs, genes from AML-associated WGCNA modules, and lipid metabolism–related genes from the REACTOME_METABOLISM_OF_LIPIDS gene set. Gene symbols were standardized to official gene symbols, and duplicated symbols were removed before intersection. This filtering strategy yielded 15 candidate genes: ACADM, CERS6, CHD9, SLC44A2, SUMO2, RAB4A, AGPAT5, MTMR2, KDSR, HADH, ACACA, RORA, MBTPS2, SUMF2, and BDH2.

To further refine these candidates, three feature-selection approaches were applied: support vector machine–recursive feature elimination (SVM-RFE), random forest (RF), and least absolute shrinkage and selection operator (LASSO) (24). LASSO regression was implemented with the glmnet package (25), using 10-fold cross-validation to select λ. Genes with nonzero coefficients were retained as LASSO-derived features. RF analysis was performed with the randomForest package (26) using 500 trees, and variables were ranked according to mean decrease in accuracy. SVM-RFE was used to iteratively remove less informative variables and identify the optimal feature set for classification. Only genes retained by all three methods were carried forward for subsequent analyses.

### 2.6 Survival analysis

The prognostic relevance of selected candidate genes was initially assessed using the KM plotter platform. AML patients were assigned to high- and low-expression groups according to the cutoff used in the KM plotter analysis, and overall survival was evaluated using the log-rank test. To further address whether MTMR2 had independent prognostic value, exploratory univariable and multivariable Cox proportional hazards regression analyses were performed using the GSE12417 cohort. The multivariable model included MTMR2 expression, age, and available FAB classification. The complete exploratory Cox regression results are provided in **Supplementary Table 1**.

### 2.7 Receiver operating characteristic analysis

The discriminatory power of candidate genes for distinguishing AML from healthy controls was examined by ROC analysis. Diagnostic capability was summarized by the area under the ROC curve (AUC).

### 2.8 Subcellular localization analysis

Subcellular localization information for MTMR2 protein was retrieved from the Human Protein Atlas (HPA) database.

### 2.9 Clinical sample collection and isolation

Bone marrow samples were collected from 16 AML patients and 9 healthy controls at Central People’s Hospital of Zhanjiang. Bone marrow mononuclear cells (BMNCs) were isolated using human lymphocyte separation medium (TBD, LTS1077) according to the manufacturer’s instructions. Clinical baseline characteristics, including body mass index (BMI), and lipid-related parameters, including total cholesterol (CHOL), apolipoprotein A1 (ApoA1), apolipoprotein B100 (ApoB100), triglycerides (TG), high-density lipoprotein cholesterol (HDL-C), and low-density lipoprotein cholesterol (LDL-C), were collected and are summarized in **Supplementary Table 2**.

### 2.10 Reverse transcription quantitative polymerase chain reaction

MTMR2 expression in clinical AML and control samples was validated by RT-qPCR. TRIzol reagent was used to isolate total RNA from BMNCs, and 1 μg of RNA was converted to cDNA using TransScript^®^ First-Strand cDNA Synthesis SuperMix (TransGen Biotech, AT301). RT-qPCR was performed on a LightCycler 480 system (Roche) using PerfectStart^®^ Green qPCR SuperMix (TransGen Biotech, AQ602). MTMR2 expression was assessed relative to GAPDH and quantified by the 2^-ΔΔCt^ method. Primer details are provided in **Supplementary Table 3**.

### 2.11 Statistical analysis

Statistical analyses were performed as described in the corresponding sections, and GraphPad Prism was used for visualization of clinical validation data. For clinical validation data, normality was assessed using the Shapiro–Wilk test. Because of the limited sample size and non-normal distribution of several variables, differences between AML patients and healthy controls were analyzed using the Mann–Whitney U test. Clinical validation data are presented as median with interquartile range unless otherwise stated. Survival differences were evaluated using the log-rank test, and exploratory Cox regression was performed as described above. Spearman correlation analysis was used to assess associations between MTMR2 expression and immune-related ssGSEA scores. P < 0.05 was considered statistically significant.

## 3 Results

### 3.1 Study workflow

**Figure 1:**
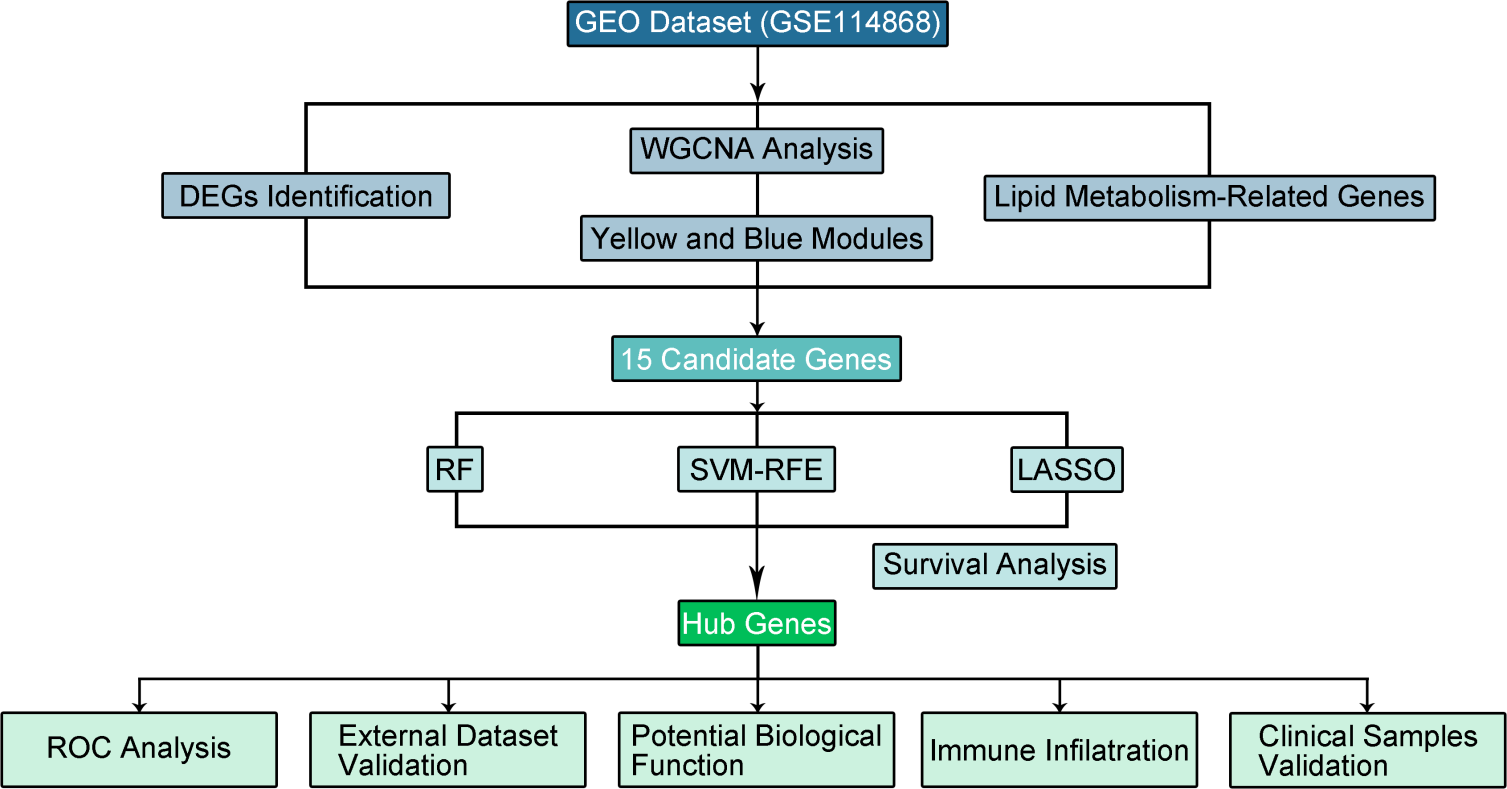
Overview of the study workflow. The GEO cohort GSE114868 served as the discovery dataset for differential expression analysis and WGCNA. Lipid metabolism–related genes were overlapped with DEGs and genes from AML-related WGCNA modules (blue and yellow), generating 15 candidate genes. These candidates were further filtered by three feature-selection approaches, including RF, SVM-RFE, and LASSO. The prioritized genes were subsequently evaluated by survival analysis, ROC analysis, external dataset validation, functional enrichment analysis, inferred immune-cell representation analysis, and RT-qPCR validation in clinical samples.

A systematic workflow was established to identify lipid metabolism-related candidate biomarkers in AML (**Figure 1**). Using GSE114868 as the discovery cohort, we first identified differentially expressed genes (DEGs) between AML and healthy control samples. Lipid metabolism-related pathway activity was evaluated using ssGSEA. WGCNA was then performed to identify AML-associated gene modules, and lipid metabolism-related candidate genes were obtained by intersecting DEGs, lipid metabolism-related genes, and WGCNA module genes. LASSO, random forest, and SVM-RFE algorithms were subsequently applied for feature selection. The prioritized genes were further evaluated by external expression validation, diagnostic ROC analysis, survival analysis, functional enrichment analysis, inferred immune-cell representation analysis, and clinical RT-qPCR validation.

### 3.2 Lipid metabolism–related transcriptional programs are elevated in AML

**Figure 2:**
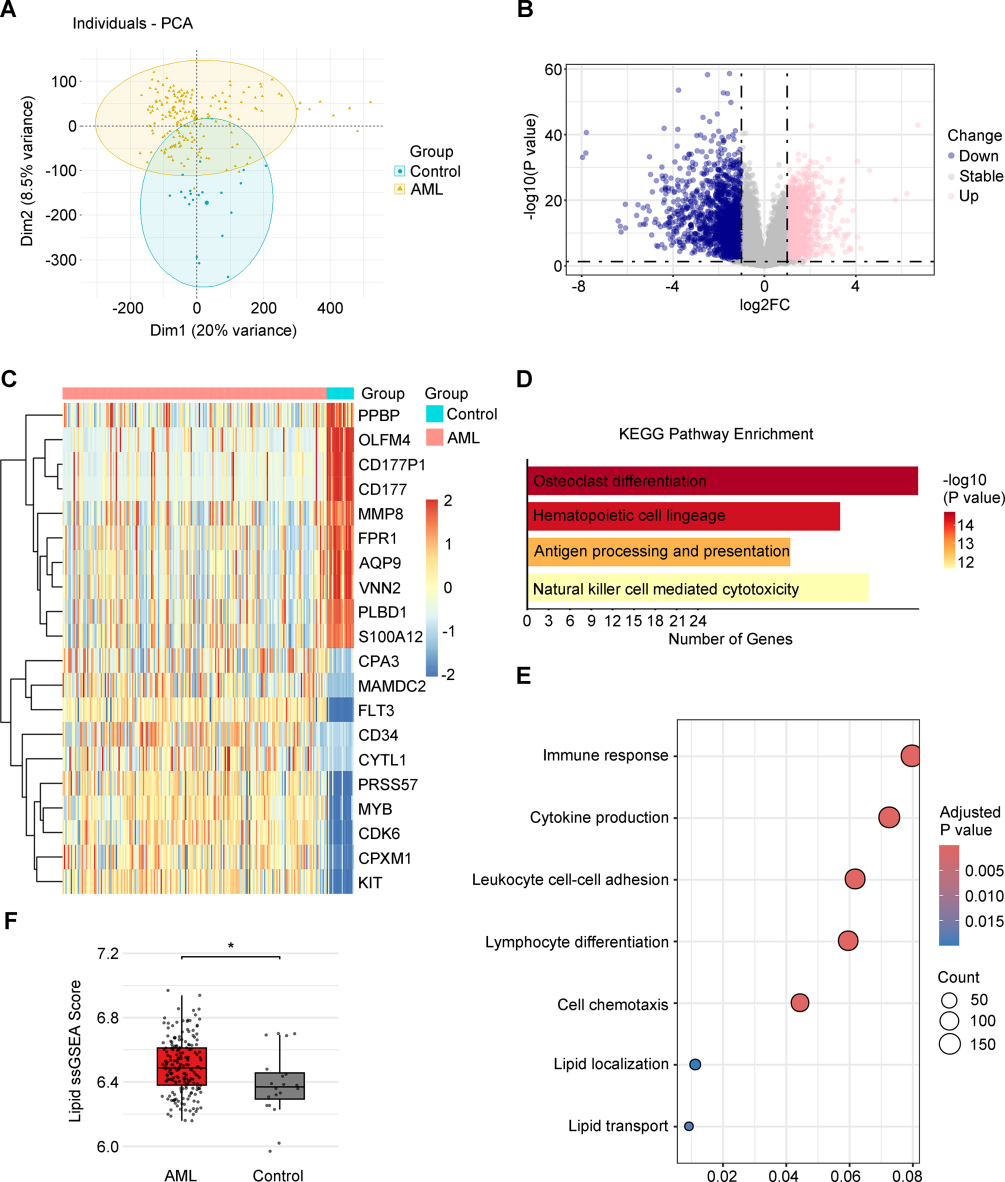
Transcriptomic differences and enrichment patterns in GSE114868. **(A)** PCA plot showing segregation of AML and healthy control samples. **(B)** Volcano plot of DEGs identified between AML and healthy control samples. **(C)** Heatmap displaying the top 10 upregulated and top 10 downregulated DEGs. **(D)** KEGG enrichment analysis of DEGs. **(E)** GO enrichment analysis of DEGs. **(F)** ssGSEA-based comparison of lipid metabolism pathway activity between AML and healthy control samples. *P < 0.05.

The GSE114868 cohort showed marked transcriptomic differences between AML and healthy control samples. PCA separated the two groups clearly, indicating distinct global expression patterns (**Figure 2A**). Using |log2FC| > 1 and adjusted P < 0.05, we identified 2,850 DEGs, including 1,304 upregulated genes and 1,546 downregulated genes in AML (**Figure 2B**). Expression patterns of the top 10 upregulated and top 10 downregulated DEGs were illustrated by heatmap (**Figure 2C**). Enrichment analyses indicated that these DEGs were mainly involved in hematopoietic cell lineage, antigen processing and presentation, immune regulation, cytokine production, leukocyte adhesion, lymphocyte differentiation, chemotaxis, and lipid localization and transport (**Figures 2D, E**). Consistent with these enrichment results, ssGSEA showed higher lipid metabolism pathway activity in AML samples than in healthy controls (**Figure 2F**).

### 3.3 Identification of AML-associated gene modules by WGCNA

**Figure 3:**
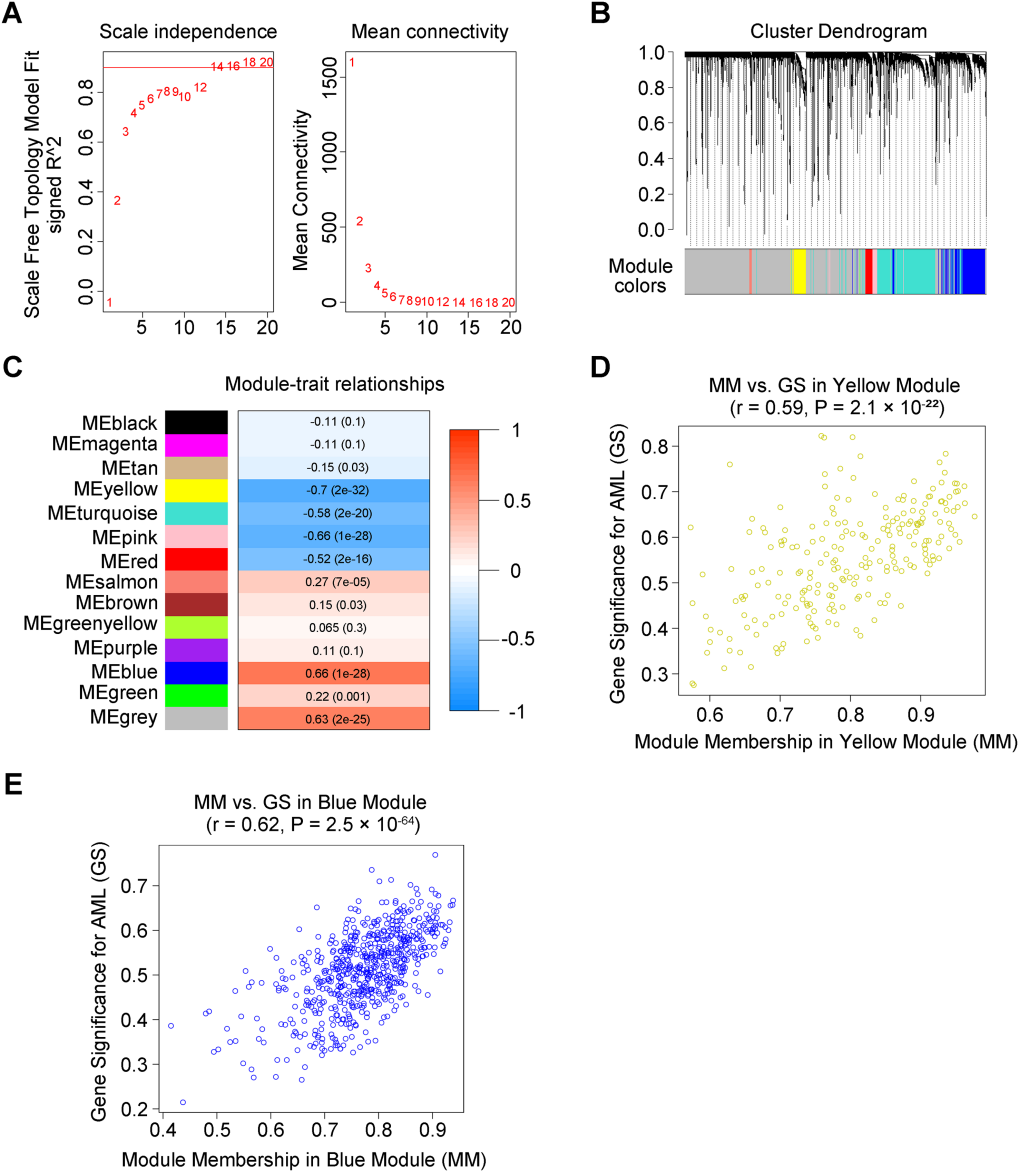
WGCNA identifies co-expression modules related to AML. **(A)** Soft-threshold selection according to scale-free topology fit index and mean connectivity. **(B)** Hierarchical clustering dendrogram of genes with module assignment indicated by color. **(C)** Heatmap showing associations between module eigengenes and AML status. **(D)** Scatter plot of MM and GS in yellow module. **(E)** Scatter plot of MM versus GS in blue module.

WGCNA of the GSE114868 expression matrix identified co-expression modules associated with AML. A soft-thresholding power of β = 14 was selected to achieve approximate scale-free topology (**Figure 3A**). Hierarchical clustering combined with dynamic tree cutting yielded 14 distinct modules (**Figure 3B**). Correlation analysis between module eigengenes and disease status showed that the yellow module was most negatively associated with AML, whereas the blue module showed the strongest positive association (**Figure 3C**). Gene significance (GS) and module membership (MM) were further examined in both modules, supporting their relevance to the AML phenotype (**Figures 3D, E**). Therefore, the blue and yellow modules were retained for subsequent analyses.

### 3.4 Machine-learning prioritization identifies four lipid metabolism–related candidate genes in AML

**Figure 4:**
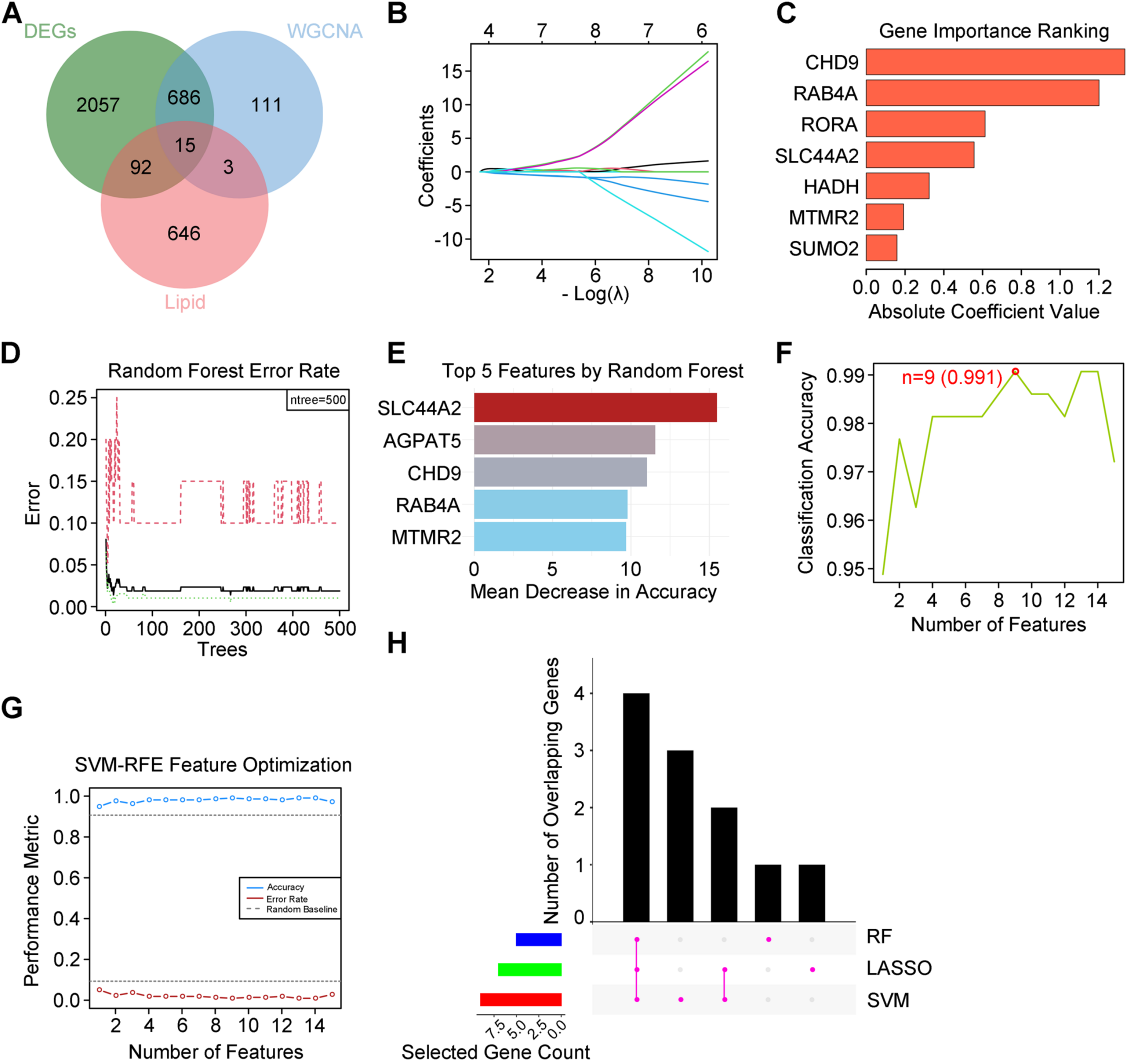
Feature selection of lipid metabolism–related candidate genes by machine-learning approaches. **(A)** Overlap of lipid metabolism–related genes, DEGs, and genes derived from AML-associated WGCNA modules, yielding 15 candidates. **(B)** LASSO coefficient trajectories across log(λ) values. **(C)** Absolute coefficient values of genes retained in the LASSO model. **(D)** Random forest error rate plotted against tree number, with ntree set to 500. **(E)** Variable importance ranking from the random forest model, shown as mean decrease in accuracy. **(F)** Classification accuracy obtained with different feature numbers. **(G)** SVM-RFE optimization results across feature counts. **(H)** UpSet plot showing genes shared by the RF, LASSO, and SVM-RFE results.

The intersection of genes from AML-associated WGCNA modules, lipid metabolism–related genes, and DEGs yielded 15 candidate genes (**Figure 4A**). These candidates were further filtered using LASSO, random forest, and SVM-RFE. LASSO regression reduced the feature set based on coefficient shrinkage across different λ values (**Figure 4B**), with CHD9 and RAB4A contributing prominently to the model according to coefficient magnitude (**Figure 4C**). In the random forest model, the error rate declined rapidly and then stabilized as the number of trees increased, indicating model stability when 500 trees were used (**Figure 4D**). Feature ranking by mean decrease in accuracy placed SLC44A2, AGPAT5, CHD9, RAB4A, and MTMR2 among the top variables (**Figure 4E**). SVM-RFE achieved optimal classification performance when nine features were retained, with an accuracy of 0.991 (**Figures 4F, G**). Comparison of the three feature-selection results identified four overlapping genes: CHD9, SLC44A2, RAB4A, and MTMR2 (**Figure 4H**).

### 3.5 MTMR2 was prioritized as a candidate biomarker in AML

**Figure 5:**
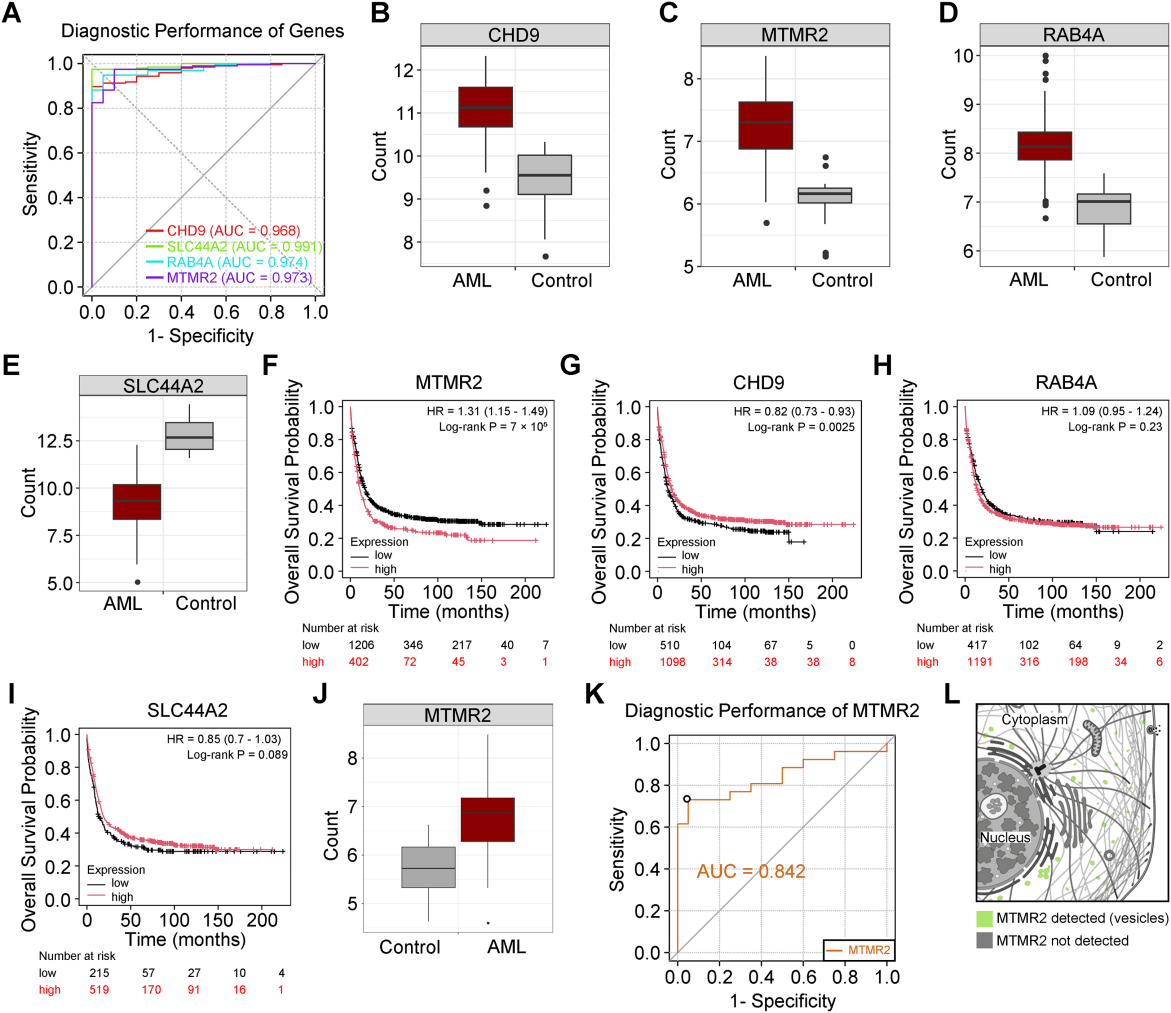
Diagnostic assessment and prioritization of MTMR2 in AML. **(A)** ROC analysis for the candidate genes in the GSE114868 cohort. **(B–E)** Expression distributions of candidate genes in AML and healthy control samples [**(B)**, CHD9; **(C)**, MTMR2; **(D)**, RAB4A; **(E)**, SLC44A2]. **(F–I)** Kaplan–Meier survival curves according to gene expression level [**(F)**, MTMR2; **(G)** CHD9; **(H)** RAB4A; **(I)** SLC44A2]. **(J)** External validation of MTMR2 expression in GSE9476. **(K)** ROC curve for MTMR2 in GSE9476. **(L)** HPA-based subcellular localization pattern of MTMR2 protein [image adapted from the Human Protein Atlas (HPA), with a Creative Commons Attribution-ShareAlike 4.0 International License (CC BY-SA 4.0)].

All four overlapping genes showed discriminatory potential for AML in the GSE114868 cohort according to ROC analysis (**Figure 5A**). Expression comparison indicated that CHD9, MTMR2, and RAB4A were upregulated in AML, whereas SLC44A2 was downregulated (**Figures 5B–E**). Kaplan–Meier survival analysis further showed that MTMR2 was the only candidate gene that combined significant overexpression in AML with an adverse survival association (**Figures 5F–I**). In the external validation cohort GSE9476, MTMR2 remained significantly elevated in AML samples relative to controls (**Figure 5J**). ROC analysis in this dataset yielded an AUC of 0.842, supporting a moderate-to-good ability of MTMR2 to distinguish AML from healthy controls (**Figure 5K**). HPA data indicated that MTMR2 protein was mainly localized to intracellular vesicular structures (**Figure 5L**). Taken together, MTMR2 was prioritized as the main candidate gene for downstream analyses.

### 3.6 MTMR2-associated transcriptional alterations are linked to immune- and inflammation-related pathways in AML

**Figure 6:**
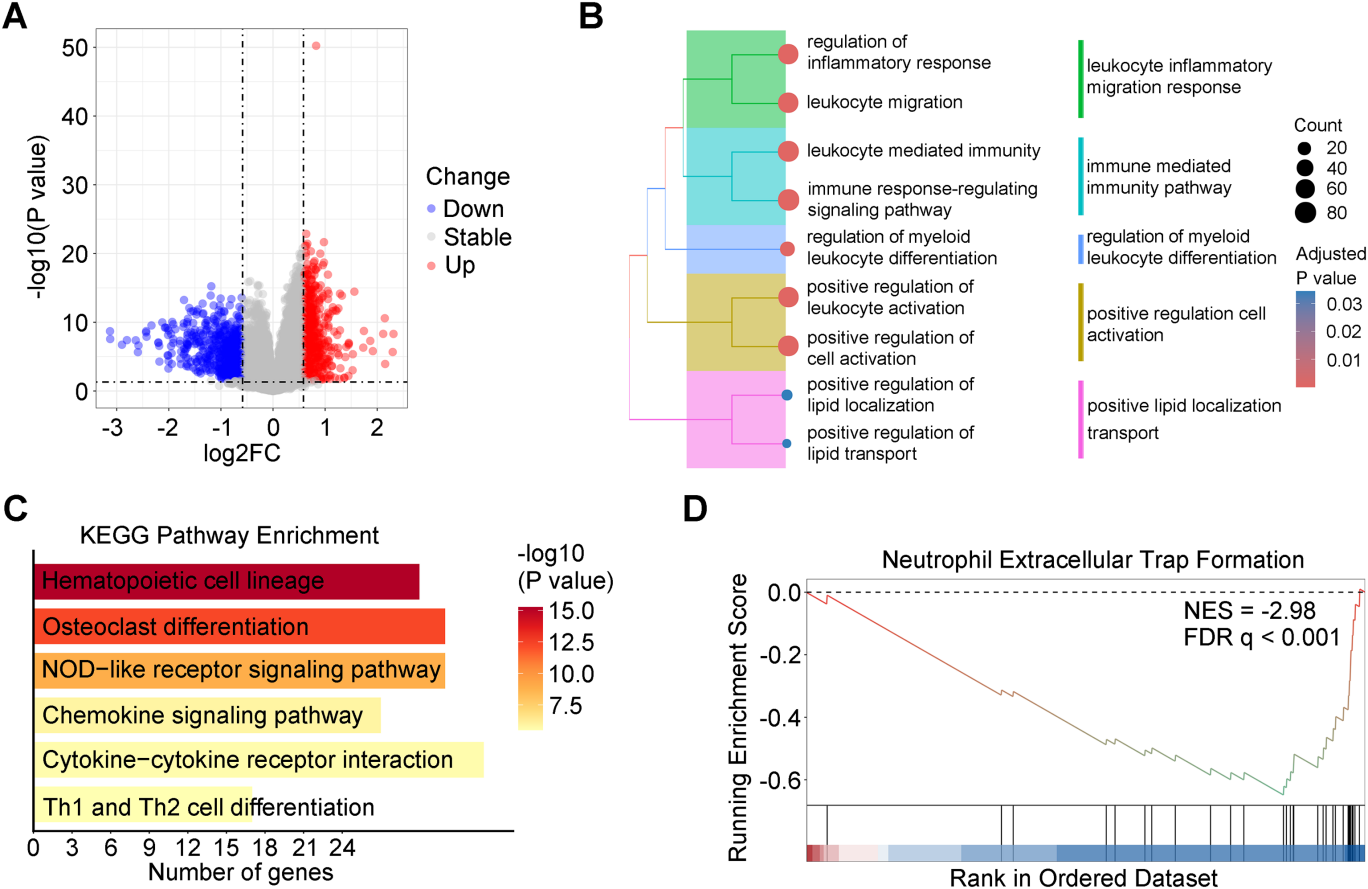
MTMR2-associated transcriptomic changes are enriched in immune- and inflammation-related pathways in AML. **(A)** Volcano plot of DEGs identified between the MTMR2-low and MTMR2-high groups. **(B)** GO analysis profile of MTMR2-associated DEGs. **(C)** KEGG enrichment profile of MTMR2-associated DEGs. **(D)** GSEA plot showing enrichment of the neutrophil extracellular trap formation signature in the MTMR2-low subgroup rather than the MTMR2-high subgroup (NES = −2.98, FDR q < 0.001).

To characterize the transcriptomic context associated with MTMR2, samples in GSE114868 were assigned to MTMR2-high and MTMR2-low subgroups using the median expression cutoff. This comparison identified 1,331 DEGs, including 610 upregulated and 721 downregulated genes in the MTMR2-high group (**Figure 6A**). GO analysis revealed enrichment of immune-related biological processes as well as lipid localization and transport (**Figure 6B**), suggesting that MTMR2-associated transcriptional alterations are linked to immune signaling and lipid-related processes. KEGG analysis highlighted hematopoietic cell lineage, osteoclast differentiation, and Th1/Th2 cell differentiation pathways (**Figure 6C**). In addition, GSEA showed that the neutrophil extracellular trap formation signature was enriched in the MTMR2-low subgroup rather than the MTMR2-high subgroup (NES = −2.98, FDR q < 0.001; **Figure 6D**).

### 3.7 MTMR2 is associated with inferred immune-cell representation in AML

**Figure 7:**
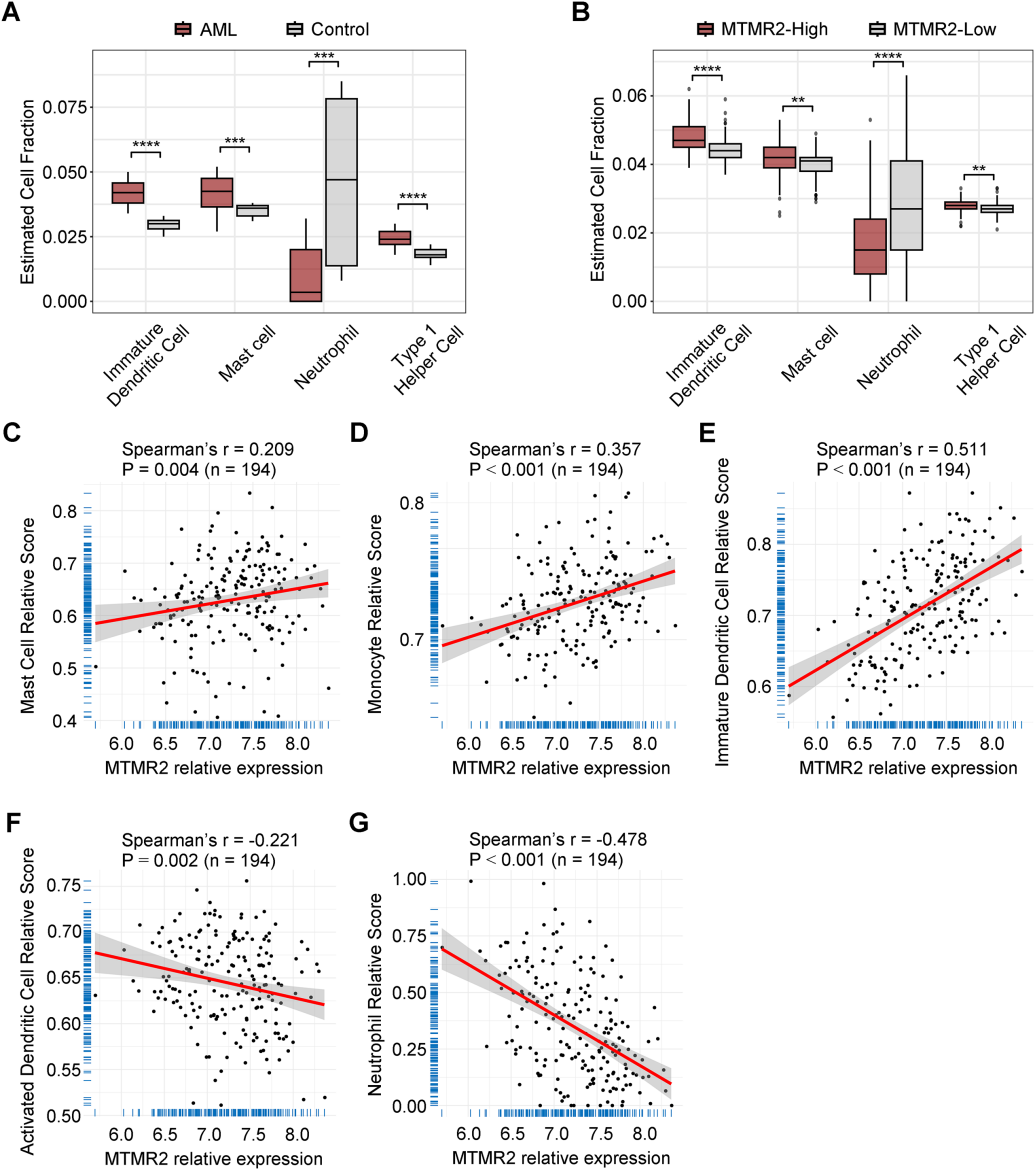
Relationship between MTMR2 expression and inferred immune-cell representation in AML. **(A)** ssGSEA-based immune-cell scores in AML and healthy control samples. **(B)** ssGSEA-based immune-cell scores in the MTMR2-low and MTMR2-high groups. **(C–G)** Spearman correlation plots between MTMR2 expression and representative immune-cell ssGSEA scores. *P < 0.05, **P < 0.01, ***P < 0.001, ****P < 0.0001.

Because MTMR2-associated DEGs were enriched in immune-related functions, we further examined immune-cell representation using ssGSEA. Compared with healthy controls, AML samples showed higher ssGSEA scores for immature dendritic cells, mast cells, and type 1 helper T cells, whereas neutrophil scores were lower (**Figure 7A**). A similar pattern was observed after stratifying AML samples by MTMR2 expression. The MTMR2-high group showed higher scores for immature dendritic cells, mast cells, and type 1 helper T cells, together with lower neutrophil scores, compared with the MTMR2-low group (**Figure 7B**). Spearman correlation analysis further showed that MTMR2 expression was positively associated with mast cells, monocytes, and immature dendritic cells, and negatively associated with activated dendritic cells and neutrophils (**Figures 7C–G**).

### 3.8 Preliminary clinical validation of MTMR2 expression and lipid-related clinical alterations in AML

**Figure 8:**
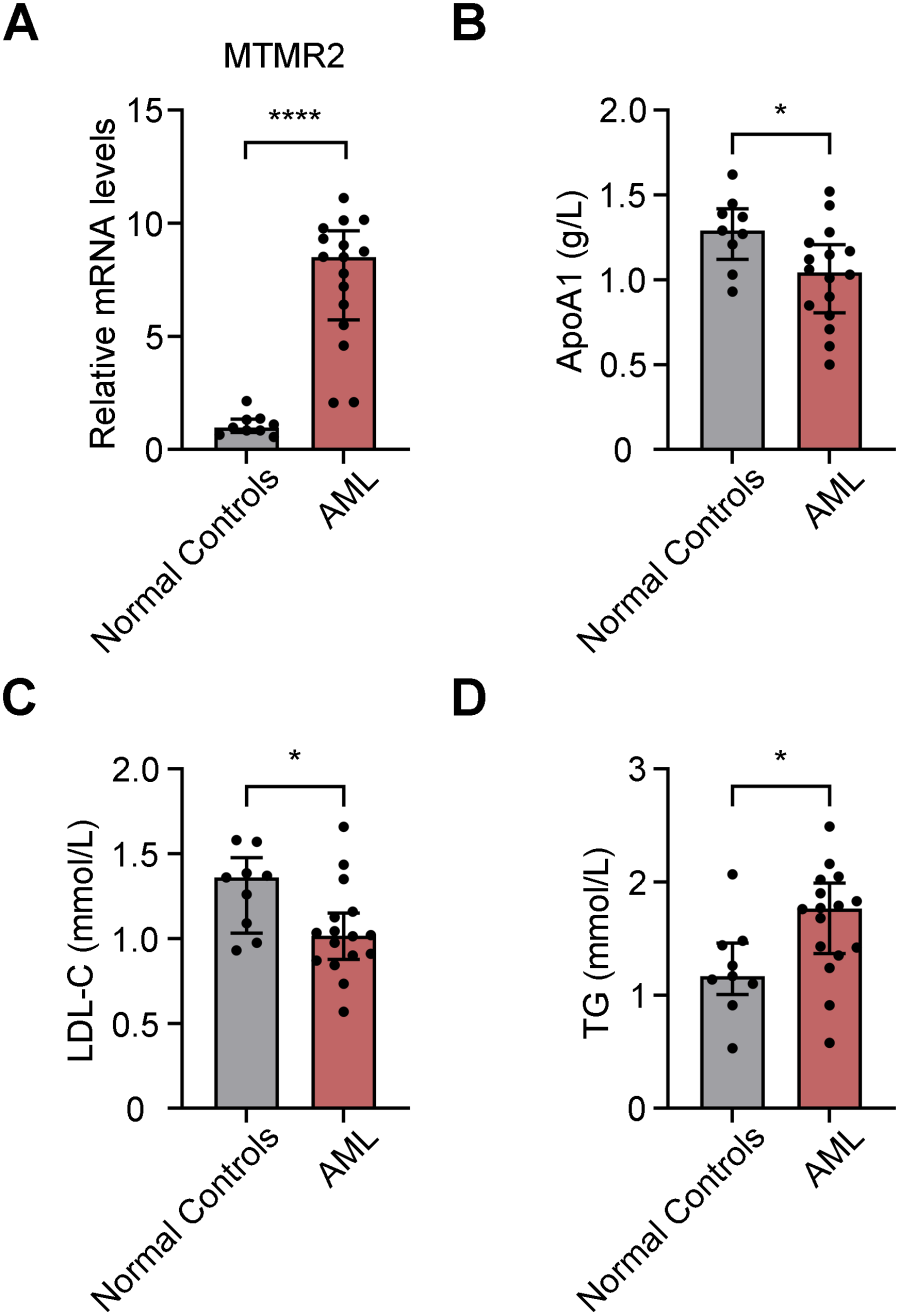
Clinical validation of MTMR2 expression and lipid-related clinical parameters in AML. **(A)** Relative MTMR2 mRNA expression was measured by RT-qPCR in AML samples and healthy controls. **(B–D)** Comparison of lipid-related clinical parameters between AML patients and healthy controls, including ApoA1 **(B)**, LDL-C **(C)**, and TG **(D)**. AML patients, n = 16; healthy controls, n = 9. Individual data points are shown. Data are presented as median with interquartile range. Normality was assessed using the Shapiro–Wilk test, and between-group differences were analyzed using the Mann–Whitney U test. *P < 0.05; ****P < 0.0001.

RT-qPCR analysis showed that MTMR2 mRNA expression was significantly higher in AML samples than in healthy controls (**Figure 8A**). This finding was consistent with the expression pattern observed in both GSE114868 and GSE9476. We further examined lipid-related clinical parameters in the same cohort. ApoA1 and LDL-C levels were lower in AML patients, whereas TG levels were higher, and these differences were statistically significant (**Figures 8B–D**). BMI, CHOL, ApoB100, and HDL-C showed no statistically significant differences between AML patients and healthy controls (**Supplementary Figure 1**).

Together, these findings provide preliminary clinical validation of increased MTMR2 expression in AML. The lipid-related clinical data further suggest that AML may be accompanied by alterations in circulating lipid-related parameters. Given the limited sample size, these results should be interpreted as exploratory and require validation in larger independent cohorts.

## 4 Discussion

### 4.1 MTMR2 was identified as a lipid metabolism–associated candidate biomarker in AML

This study used an integrative bioinformatic framework to screen for lipid metabolism–related candidate biomarkers in AML. Transcriptomic analyses indicated increased lipid metabolism–related activity in AML, and machine-learning-based feature selection narrowed the candidate list to four genes. Among these candidates, MTMR2 was prioritized for further investigation because it showed moderate-to-good diagnostic performance and a Kaplan–Meier-based adverse survival association. Its upregulation in AML was further supported by external validation in an independent GEO cohort and by RT-qPCR analysis of clinical samples. Collectively, these findings support MTMR2 as a lipid metabolism–associated candidate biomarker in AML.

Our observations are consistent with increasing evidence that metabolic reprogramming contributes to AML biology. Although glucose and amino acid metabolism have long been emphasized, recent studies have increasingly highlighted lipid metabolism as an important determinant of leukemic cell survival, metabolic adaptation, and therapeutic vulnerability (1, 3, 27–29). Lipid metabolic remodeling has been implicated in membrane synthesis, signaling regulation, redox balance, and treatment resistance in AML, providing a biological rationale for focusing on lipid metabolism–related genes (3). Therefore, the elevated lipid metabolism pathway activity observed in our study is biologically plausible and consistent with current understanding of AML metabolism (1, 3).

### 4.2 Potential biological relevance of MTMR2 in AML

MTMR2 is of biological interest because it belongs to the myotubularin family of phosphoinositide phosphatases and has enzymatic activity toward phosphatidylinositol 3-phosphate and phosphatidylinositol 3,5-bisphosphate (17). Previous studies have also localized MTMR2 to endosomal structures and implicated it in phosphoinositide-regulated membrane trafficking (17). Given the close links between phosphoinositide metabolism, membrane dynamics, intracellular signaling, and vesicle-associated processes, the association between MTMR2 and lipid metabolism–related programs observed in our study is biologically plausible. However, our data do not determine whether MTMR2 directly regulates leukemic metabolism or merely reflects a broader AML-associated transcriptional state.

A further important observation was the close association between MTMR2 and immune-related transcriptional programs. Pathway analyses of MTMR2-associated genes highlighted enrichment in immune- and inflammation-related processes, and GSEA further showed negative enrichment of the neutrophil extracellular trap formation signature in the MTMR2-high phenotype. Elevated MTMR2 expression was also associated with inferred immune-cell representation characterized by higher ssGSEA scores for immature dendritic cells, mast cells, and monocytes, together with lower scores for activated dendritic cells and neutrophils. These findings are notable because AML is increasingly recognized as a disease characterized by profound immune dysregulation, in which leukemic cells and dysfunctional immune components jointly contribute to immune evasion and disease persistence (9, 10). Therefore, our results suggest that MTMR2 may mark a transcriptional state linked not only to lipid metabolism-related alterations but also to immune-related biological programs in AML. Nevertheless, because the immune analyses in this study were inference-based, these findings should be interpreted as associations rather than direct evidence of immunomodulatory activity.

From a translational perspective, MTMR2 may have potential relevance as a candidate biomarker reflecting metabolic and immune-related transcriptional states in AML. Despite advances in molecular classification and targeted treatment, AML remains highly heterogeneous, and relapse continues to be a major clinical challenge (30). In this study, MTMR2 showed reproducible upregulation across datasets and clinical samples and was associated with inferior overall survival in Kaplan–Meier analysis. These findings suggest potential clinical relevance, although the prognostic value of MTMR2 requires further validation in larger cohorts with complete clinical and molecular annotations.

### 4.3 Current limitations and future research directions

Several limitations should be acknowledged. First, this study was mainly based on public transcriptomic datasets and correlation-based analyses. Therefore, the identified associations among MTMR2 expression, lipid metabolism-related signatures, and immune-related features do not establish direct causality.

Second, although KM plotter analysis suggested an adverse survival association for high MTMR2 expression, exploratory Cox regression using the GSE12417 cohort did not support MTMR2 as an independent prognostic factor after adjustment for available clinical covariates, including age and FAB classification (**Supplementary Table 1**). Therefore, the survival-related findings should be interpreted as Kaplan–Meier-based associations rather than evidence of independent prognostic value. In addition, because GSE12417 is a cytogenetically normal AML cohort profiled on multiple Affymetrix microarray platforms and the publicly available annotations are insufficient for comprehensive adjustment for established AML prognostic variables, the absence of independent prognostic value for MTMR2 should be re-evaluated in larger, well-annotated AML cohorts with standardized treatment information and comprehensive molecular risk stratification.

Third, although the clinical validation cohort was expanded and lipid-related clinical parameters were added, the sample size remained limited, and complete molecular risk stratification, treatment information, and follow-up data were unavailable.

Finally, functional experiments were not performed in this study. Future studies should establish MTMR2 loss- and gain-of-function models in AML cell lines and, where feasible, patient-derived AML cells. These models could be used to examine whether MTMR2 affects leukemic cell proliferation, apoptosis, cell-cycle progression, adhesion/migration, and drug response. Lipid-related assays, including lipid droplet staining, fatty acid uptake or oxidation analysis, phosphoinositide-related signaling or endosomal trafficking analysis, and lipidomic profiling, would help determine whether MTMR2 is involved in AML-associated lipid metabolic remodeling. Rescue experiments would further clarify whether MTMR2 functionally contributes to these phenotypes or mainly reflects a broader AML-associated transcriptional state.

## Data Availability

The datasets presented in this study can be found in online repositories. The names of the repository/repositories and accession number(s) can be found in the article/**Supplementary Material**.

## Supplementary material

Supplementary Figure 1 Comparison of BMI and additional lipid-related clinical parameters between AML patients and healthy controls. **(A–D)** Comparison of BMI **(A)**, CHOL **(B)**, ApoB100 **(C)**, and HDL-C **(D)** between AML patients and healthy controls. AML patients, n = 16; healthy controls, n = 9. Individual data points are shown. Data are presented as median with interquartile range. Normality was assessed using the Shapiro–Wilk test, and between-group differences were analyzed using the Mann–Whitney U test. ns, not significant.
