# Supplementary material for: Identification of MTMR2 as an AML-associated candidate biomarker derived from lipid metabolism–related transcriptomic analysis
Source: Front Oncol. 2026 May 29;16:1837038. doi: 10.3389/fonc.2026.1837038 (PMC13259702; doi:10.3389/fonc.2026.1837038)
Supplement: Supplementary Figure 1 — Comparison of BMI and additional lipid-related clinical parameters between AML patients and healthy controls. (A–D) Comparison of BMI (A), CHOL (B), ApoB100 (C), and HDL-C (D) between AML patients and healthy controls. AML patients, n = 16; healthy controls, n = 9. Individual data points are shown. Data are presented as median with interquartile range. Normality was assessed using the Shapiro–Wilk test, and between-group differences were analyzed using the Mann–Whitney U test. ns, not significant. [file SupplementaryFile1.pdf]

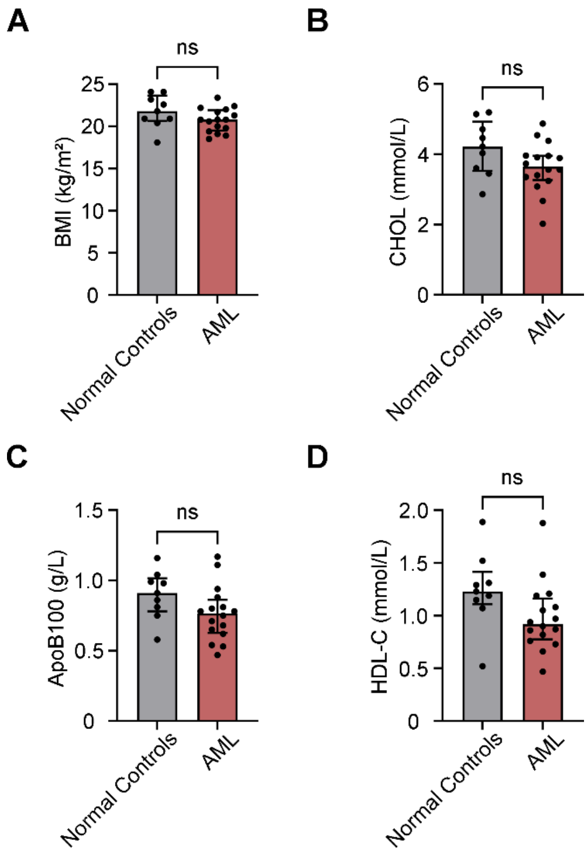

Figure S1. Comparison of BMI and additional lipid-related clinical parameters between AML patients and healthy controls.

(A–D) Comparison of BMI (A), CHOL (B), ApoB100 (C), and HDL-C (D) between AML patients and healthy controls. AML patients, n = 16; healthy controls, n = 9. Individual data points are shown. Data are presented as median with interquartile range. Normality was assessed using the Shapiro–Wilk test, and between-group differences were analyzed using the Mann–Whitney U test. ns, not significant.
